# COVID-19 vaccination and venous thromboembolism risk in older veterans

**DOI:** 10.1017/cts.2022.527

**Published:** 2023-02-01

**Authors:** Peter L. Elkin, Steven H. Brown, Skyler Resendez, Wilmon McCray, Melissa Resnick, Kendria Hall, Gillian Franklin, Jean M. Connors, Mary Cushman

**Affiliations:** 1 Department of Biomedical Informatics, University at Buffalo, NY, USA; 2 Department of Veterans Affairs, Knowledge based Systems and WNY VA, USA; 3 Hematology Division Brigham and Women’s Hospital, Harvard Medical School, Boston, MA, USA; 4 Larner College of Medicine at the University of Vermont, Burlington, VT, USA

**Keywords:** Vaccination, venous thromboembolism, EHR data, informatics, data science

## Abstract

**Introduction:**

It is important for SARS-CoV-2 vaccine providers, vaccine recipients, and those not yet vaccinated to be well informed about vaccine side effects. We sought to estimate the risk of post-vaccination venous thromboembolism (VTE) to meet this need.

**Methods:**

We conducted a retrospective cohort study to quantify excess VTE risk associated with SARS-CoV-2 vaccination in US veterans age 45 and older using data from the Department of Veterans Affairs (VA) National Surveillance Tool. The vaccinated cohort received at least one dose of a SARS-CoV-2 vaccine at least 60 days prior to 3/06/22 (N = 855,686). The control group was those not vaccinated (*N* = 321,676). All patients were COVID-19 tested at least once before vaccination with a negative test. The main outcome was VTE documented by ICD10-CM codes.

**Results:**

Vaccinated persons had a VTE rate of 1.3755 (CI: 1.3752–1.3758) per thousand, which was 0.1 percent over the baseline rate of 1.3741 (CI: 1.3738–1.3744) per thousand in the unvaccinated patients, or 1.4 excess cases per 1,000,000. All vaccine types showed a minimal increased rate of VTE (rate of VTE per 1000 was 1.3761 (CI: 1.3754–1.3768) for Janssen; 1.3757 (CI: 1.3754–1.3761) for Pfizer, and for Moderna, the rate was 1.3757 (CI: 1.3748–1.3877)). The tiny differences in rates comparing either Janssen or Pfizer vaccine to Moderna were statistically significant (*p* < 0.001). Adjusting for age, sex, BMI, 2-year Elixhauser score, and race, the vaccinated group had a minimally higher relative risk of VTE as compared to controls (1.0009927 CI: 1.007673–1.0012181; *p* < 0.001).

**Conclusion:**

The results provide reassurance that there is only a trivial increased risk of VTE with the current US SARS-CoV-2 vaccines used in veterans older than age 45. This risk is significantly less than VTE risk among hospitalized COVID-19 patients. The risk-benefit ratio favors vaccination, given the VTE rate, mortality, and morbidity associated with COVID-19 infection.

## Introduction

As of January 3, 2022, over 826,000 people in the United States of America (USA) have died from COVID-19, and worldwide deaths exceed 5.45 million [1]. SARS-CoV-2 vaccine administration in the USA began in December 2020. To date, in the USA over 245 million people have been vaccinated, over 204 million are fully vaccinated, and 68 million people have received booster vaccines [2]. Worldwide, 9.23 billion doses of vaccine have been administered – a rate of 120 doses per 100 people.

In the USA, there are 3 vaccines for the prevention of COVID-19 with Emergency Use Authorization or approval: Pfizer-BioNTech BNT162b2, approved on August 23, 2021 [3]; Moderna mRNA-1273 (authorized 12/18/20) [4] and Janssen Ad26.COV2.S, authorized 2/27/21 [5].

In order to receive Emergency Use Authorization from the US Food and Drug Administration (FDA), companies were required to conduct phase 3 studies to determine vaccine efficacy, monitor adverse reactions, and submit the results to the FDA for review [6]. Results of the initial trials showed no signal of increased risk of deep vein thrombosis (DVT) or pulmonary embolism (PE) with administration. Pfizer monitored adverse reactions in 21,720 vaccine recipients and 21,728 placebo recipients [7]. DVT or PE occurred in 1 participant each with vaccine and 1 each with placebo. In their phase 3 study, Moderna monitored 15,185 vaccine recipients; two had DVT, and four had PE. Of 15,166 placebo recipients, 0 had DVT and five had PE [8]. The Janssen phase 3 study included 21,895 vaccine recipients and 21,888 placebo recipients [9,10]; over 28 days DVT occurred in five vaccine recipients and two with placebo, while PE occurred in two vaccinated participants and one with placebo. A recent report from Minnesota suggested no increased risk of VTE with these vaccines [11].

The recognition of a syndrome of vaccine-induced thrombotic thrombocytopenia (VITT) was first seen in the US after 6.8 million people received the Janssen vaccine [11]. VITT is now recognized as a rare complication of adenoviral vector-based SARS-CoV-2 vaccines, including the Janssen vaccine [12–14]. In December 2021, the CDC issued interim clinical considerations for SARS-CoV-2 vaccine use, stating a preference for mRNA vaccines [12].

Although VITT is rare, it is plausible that less severe cases may have not been recognized. It is also possible that VITT and VTE were underreported to the US Vaccine Adverse Events Reporting System, a well-documented phenomenon [13,14]. Concern about VITT or VTE after these vaccines might contribute to vaccine hesitancy [15]. To provide additional information, we investigated VTE rates after receiving the Pfizer, Moderna, and Janssen vaccines in the US Department of Veterans Affairs (VA) population aged >45 years. The incidence rates for VTE in the general population >age 45 are 1000-fold higher than younger groups. [16,17]. This makes the results of our study relevant to this at-risk population.

## Methods

We used data from the Department of VA Corporate Data Warehouse (CDW) National Surveillance Tool (NST). The VA CDW is a relational database with over 60 clinical domains from all 128 VA Veterans Information System Technology Architecture systems (VistA) [18,19]. CDW is updated nightly and used for a variety of purposes in the VA including management, reporting, clinical and administrative research, and quality improvement. The VA developed the NST during the COVID-19 pandemic to be the authoritative VA data source for outbreaks [20]. NST clinical data sources include inpatient Admission Discharge Transfer records, outpatient encounters, clinicians’ notes, orders, labs, and medications. We accessed VA COVID-19 vaccination, diagnosis, and laboratory data through the VA Health Services Research and Development VA Informatics and Computing Infrastructure mirror of the production NST [21].

The study period began 1/1/2020 and ended 3/06/22. All persons 45 years of age or older who received at least one dose of a SARS-CoV-2 vaccine at least 60 days prior to and including 3/06/22 were included in the vaccinated cohort. All vaccinated patients were examined for a VTE event within 60 days of receiving their vaccination. The comparison (control) group included veterans 45 years of age and older, who were unvaccinated. We selected this age range as this limitation was sufficient to equalize the age ranges in the vaccinated and unvaccinated groups. Age is a known independent risk factor for VTE. All patients in the study never had a positive COVID-19 test. Patients in the vaccinated group were evaluated over a sixty-day period after the date of the first vaccination. All patients in both groups were COVID-19 tested at least once. The controls were COVID-19 negative for all tests, to eliminate from the controls COVID-19 related VTEs. Patients who had a VTE within six months prior to their vaccination date (vaccination group) or the start of the control period (control group) were excluded. Members of the vaccination and control groups were alive throughout the evaluation period.

The main outcome was the development of a VTE within 60 days of the first vaccine administration. In the control group, we identified VTE events that occurred for the 60-day period from 11/02/21 to 12/31/21. VTE was defined by the presence of any of the following ICD10-CM codes:

- DVT: I82.40; thigh I82.4y, DVT femoral I82.41; DVT iliac (iliofemoral) I82.42 DVT popliteal I82.43;
- cavernous sinus venous thrombosis: I67.6;
- portal vein thrombosis / embolism: I81;
- PE: pulmonary (acute) (artery) (vein) I26.99;

All inpatient ICD10 codes are validated by health information management personnel, and 20% of outpatient diagnoses are audited as is generally done for all VA cases. We tabulated the counts of VTE overall, and by VTE type, and the incidence rate per thousand participants. We calculated the odds ratio of VTE (and VTE type) for each vaccine compared to no vaccine using logistic regression. We assessed subtypes of VTE between the vaccinated group and controls using chi-square testing. We performed multivariable regression analyses adjusted for age, sex, race, ethnicity, BMI, and 2-year Elixhauser Scores of comorbidities to account for potential confounding. We then calculated odds ratios for the Janssen vaccine compared to the Moderna and Pfizer vaccines. We used analysis of variance to understand the co-variance between the different variables in our model.

## Results

The study included 855,686 vaccinated patients and 321,676 unvaccinated patients. The demographic characteristics of each group according to vaccine type are shown in Table 1. Of the 855,686 vaccine recipients, 388,920 received the Pfizer vaccine, 417,183 received the Moderna vaccine, and 49,583 received the Janssen vaccine. Vaccine recipients were younger than controls. Janssen vaccine recipients were younger than Moderna and Pfizer vaccine recipients.

**Table 1.** Demographics of the vaccinated group and controls

Characteristic (mean, 95% CI, or %, number	Controls	Vaccinated	Janssen	Pfizer	Moderna
Age	62.48 (CI: 62.45 - 62.52)	67.00 (CI: 66.98 – 67.02)	62.85 (CI: 62.77 – 62.94)	66.66 (CI: 66.63 – 66.69)	67.82 (CI: 67.79 – 67.85)
Male	75.5% (242,755)	91.1% (779,555)	89.9% (44,583)	90.4% (351,650)	91.9% (383,322)
Ethnicity – Hispanic	4.44% (14,280)	7.24% (61,981)	5.94% (2,945)	6.89% (26,814)	7.72% (32,222)
Race					
American Indian	0.65% (2,082)	0.70% (6,004)	0.82% (408)	0.69% (2,679)	0.70% (2,917)
Black	15.92% (51,218)	23.46% (200,713)	21.28% (10,550)	27.77% (107,989)	19.70% (82,174)
Pacific Islander/Hawaiian	0.65% (2,088)	0.84% (7,169)	0.79% (394)	0.88% (3,434)	0.80% (3,341)
White	56.29% (181,061)	67.81% (580,215)	69.68% (34,551)	63.24% (245,961)	71.84% (299,703)
Asian	0.63% (2,042)	0.98% (8,344)	0.79% (392)	1.02% (3,958)	0.96% (3,994)
Unknown	25.86% (83,185)	6.22% (53,241)	6.63% (3,288)	6.40% (24,899)	6.01% (25,054)

As shown in Table 2, there were 442 VTE events within a 60-day period from 11/02/21 to 12/31/21 in the control group, and 2,388 VTE events in the vaccinated group within 60 days of their first vaccination. The vaccinated group had an adjusted VTE rate of 1.3755 per thousand over 60 days, which was higher than the rate in the unvaccinated group; 1.3741 per thousand (*p* < 0.001). These were adjusted by race, ethnicity, sex, age, BMI, and 2-year Elixhauser scores.

**Table 2.** Venous thromboembolism (VTE) rates by vaccine type and controls

	Total Cases	No VTE	VTE	Univariate VTE Rate Per 1000	Multivariate VTE Rate Per 1000	Multivariate Confidence Intervals	Multivariate Excess Rate per 1,000,000
Controls	321,676	321,234	442	1.3741	1.3741	1.3738-1.3744	
Vaccine	855,686	853,289	2,388	2.7907	1.3755	1.3752-1.3758	1.4
Janssen	49,583	49,434	149	3.0051	1.3761	1.3754-1.3768	2.0
Pfizer	388,920	387,752	1,168	3.0032	1.3757	1.3754-1.3761	1.6
Moderna	417,183	416,113	1,071	2.5672	1.3751	1.3748-1.3877	1.0

The rates were from the multivariate regression analysis and their 95% confidence intervals. The regression analysis was adjusted by age, race, ethnicity, sex, BMI, and 2-year Elixhauser score.

Vaccinated patients had an increased VTE rate of 0.10 percent over the multivariable-adjusted baseline rate of 1.3741 (CI: 1.3738–1.37544) VTE per thousand in the unvaccinated patients; the vaccinated rate was 1.3755 (CI: 1.3752–1.3758) or 1.4 excess cases per 1,000,000. Janssen and Pfizer vaccine types showed a minimal increased risk for VTE (Janssen rate of VTE was 1.3761 (CI: 1.3754–1.3768); Moderna rate was 1.3751 (CI: 1.3748–1.3877); Pfizer VTE rate was 1.3757 (CI: 1.3754–1.3761) all in rate per 1000 cases). The Janssen vaccine VTE rate and the Pfizer rate were significantly higher than the Moderna vaccine VTE rate (*p* < 0.001), with no other significant differences among vaccines.

Most thrombotic events were DVT and/or PE (94.5% in the vaccinated group and 95.7% in the unvaccinated group) (see Table 3). Recipients of the Janssen vaccine had higher rates of portal vein thrombosis than either Pfizer or Moderna vaccine recipients or the controls; however, these events, along with CVST, were uncommon.

**Table 3.** Thrombotic event rates (deep venous thrombosis (DVT), pulmonary embolism (PE), central venous sinus thrombosis (CVST))

	Controls		Any Vaccine		Janssen		Pfizer		Moderna	
Thrombosis Type	Case Count	Rate per 1000	Case Count	Rate per 1000	Case Count	Rate per 1000	Case Count	Rate per 1000	Case Count	Rate per 1000
DVT / PE	423	1.3150	2,257	2.6376	142	2.8639	1,107	2.8463	1,008	2.4162
CVST	1	0.0031	10	0.0117	1	0.0202	5	0.0129	4	0.0096
Splanchnic Thrombosis	18	0.0560	121	0.1414	6	0.1210	56	0.1440	59	0.1414
Total	442	1.3741	2,388	2.7907	149	3.0051	1,168	3.0032	1,071	2.5672

Adjusting for age, sex, ethnicity, BMI, 2-year Elixhauser score, and race, the vaccinated group had a minimally higher relative risk of VTE as compared to controls (1.0009927 CI: 1.000767–1.0012181). Hispanic and Asian subgroups had lower relative risk compared with Caucasians. This minimally increased risk did differ substantially by vaccine type; Janssen and Pfizer had significantly more risk than Moderna of VTE (*p* < 0.001). The comparison of the Pfizer and Janssen vaccines with VTE was not statistically different. Age, gender, BMI, and 2-year Elixhauser scores were all highly significant risk factors for VTE Not Hispanic, or Latino showed a significant risk of VTE (*p* = 0.0019).

## Discussion

In this study of US veterans aged 45 and older, we observed a minimal increase in VTE risk after SARS-CoV-2 vaccination of 0.10% or 1.4 excess cases per 1,000,000. This was true for both mRNA and adenoviral vector vaccines, although VTE risk appeared to be trivially lower with the Moderna vaccine than with Pfizer or Janssen vaccines (*p* < 0.001).

Vaccination prevents severe COVID-19 and in that way decreases VTE events. A rough estimation follows. VTE is a prominent sequela of infection, occurring in about 8% of hospitalized patients and 22.7% of patients in the ICU. (22) The CDC ensemble model forecast 257,125 new COVID-19 cases and 40,039 new hospitalizations in the 28 days following 6/21/2021 [23]. This suggests at least 3203 COVID-19-related VTE cases would have occurred in that period. If all 257,125 new cases were vaccinated instead, 0.36 excess VTE cases could be expected, based on our observed rate of 1.4 excess cases per 1,000,000 vaccinated, a 99.9% lower rate.

Our study can be compared to other recent reports of vaccination and VTE risk. A nationwide study in France among those aged 75 and older found no increase in PE over 14 days after vaccination with the Pfizer vaccine [24]. Administrative data from Clalit Health Services in Israel involving 884,828 vaccinated persons with median age of 38 did not demonstrate an increased risk of PE or other thromboses (composite of arterial embolism and thrombosis, venous embolism and thrombosis, vascular insufficiency of the intestine, portal vein thrombosis, or cranial venous sinus thrombosis) with the Pfizer vaccine [25]. Klein *et al*. also found a small increase in the VTE rate. Houghton *et al* reported no increased risk of VTE from vaccination in the Mayo Clinic enterprise population of younger primarily white patients 11 without controlling for COVID-19 infection [11]. Vaccinated individuals served as their own controls.

This study has strengths and limitations. The VA CDW and NST contain normalized clinical data from all VA facilities and clinics (numbering in the thousands) on millions of veterans; however, all veterans are not included. The veteran population is overwhelmingly male (89%) and older [26]. The largest veteran age group in 2021 includes veterans between 70 and 74 years; therefore, our results may not be generalizable to younger populations and women, particularly young women. We were not able to study VITT, although it is extremely rare with vaccines used in the USA.

The rate of the unknown race was significantly higher in the control group in than the vaccine group. It is unclear why people who did not get vaccinated were less likely to report their race. All patients in the study received VA healthcare services and vaccines were widely offered. We can only speculate that there may be underlying trust issues impacting both behaviors. Further research should be conducted to determine if our speculation is correct and if failure to report race is potentially predictive of vaccine hesitancy and other future health behaviors.

Limitations include the possibility that veterans who received VA vaccinations were treated for VTE outside of the VA and that data were not available for our study. It is also possible that veterans diagnosed with VTE were vaccinated outside of the VA and that vaccination was not documented in the NST, although the VA makes a strong effort to collect vaccination data. Like any study using administrative data, VTE events could be misclassified, and this would bias findings to the null. Detection bias of events by clinicians is also possible; however since there was not an appreciation that vaccines might cause thrombosis during the observation period, we believe that detection bias was not operative. Additionally, we could not ascertain whether cases of VITT were identified.

In conclusion, these results provide reassurance that there is only a trivial increased risk of VTE with the current US COVID-19 vaccines used in veterans older than age 45. This risk is substantially less than VTE risk amongst hospitalized COVID-19 patients or with other triggering risk factors for VTE. The net clinical benefit favors vaccination and vaccination should be encouraged. Although the risk of VTE with vaccination was trivial, we suggest clinicians advise patients to be alert for signs such as a swollen leg, chest pain, or dizziness and other signs and symptoms of VTE and encourage them to seek immediate medical attention should any occur.
